# CCL18 in a Multiplex Urine-Based Assay for the Detection of Bladder Cancer

**DOI:** 10.1371/journal.pone.0037797

**Published:** 2012-05-21

**Authors:** Virginia Urquidi, Jeongsoon Kim, Myron Chang, Yunfeng Dai, Charles J. Rosser, Steve Goodison

**Affiliations:** 1 Cancer Research Institute, M.D. Anderson Cancer Center Orlando, Orlando, Florida, United States of America; 2 Department of Biostatistics, The University of Florida, Gainesville, Florida, United States of America; 3 Section of Urologic Oncology, MD Anderson Cancer Center Orlando, Orlando, Florida, United States of America; 4 Nonagen Bioscience Corp, Orlando, Florida, United States of America; University of Campinas, Brazil

## Abstract

The early detection of bladder cancer (BCa) is pivotal for successful patient treatment and management. Through genomic and proteomic studies, we have identified a number of bladder cancer-associated biomarkers that have potential clinical utility. In a case-control study, we examined voided urines from 127 subjects: 64 tumor-bearing subjects and 63 controls. The urine concentrations of the following proteins were assessed by enzyme-linked immunosorbent assay (ELISA); C-C motif chemokine 18 (CCL18), Plasminogen Activator Inhibitor 1 (PAI-1) and CD44. Data were compared to a commercial ELISA-based BCa detection assay (BTA-Trak©) and voided urinary cytology. We used analysis of the area under the curve of receiver operating characteristic curves to compare the ability of CCL18, PAI-1, CD44, and BTA to detect BCa in voided urine samples. Urinary concentrations of CCL18, PAI-1, and BTA were significantly elevated in subjects with BCa. CCL18 was the most accurate biomarker (AUC; 0.919; 95% confidence interval [CI], 0.8704-0.9674). Multivariate regression analysis highlighted CCL18 (OR; 18.31; 95% CI, 4.95-67.70, *p*<0.0001) and BTA (OR; 6.43; 95% CI, 1.86-22.21, *p* = 0.0033) as independent predictors of BCa in voided urine samples. The combination of CCL18, PAI-1 and CD44 improved the area under the curve to0.938. Preliminary results indicate that CCL18 was a highly accurate biomarker for BCa detection in this cohort. Monitoring CCL18 in voided urine samples has the potential to improve non-invasive tests for BCa diagnosis. Furthermore using the combination of CCL18, PAI-1 and CD44 may make the model more robust to errors to detect BCa over the individual biomarkers or BTA.

## Introduction

The early detection of bladder cancer (BCa) significantly improves the probability of successful patient treatment and management. The development of molecular assays that can diagnose the disease accurately, or that can augment current methods of evaluation, would be a significant advance. A molecular assay that was applicable to non-invasively obtained body fluids, would facilitate not only diagnosis of at risk patients, but also asymptomatic screening, monitoring disease recurrence and response to treatment. The advent of advanced proteomics and genomics technologies, and associated bioinformatics development, is bringing these goals into focus.

The gold standard for diagnosing BCa involves cystoscopic examination of the bladder along with biopsy and pathologic evaluation of a bladder lesion. Voided urinary cytology (VUC) remains as the go-to non-invasive adjunct to cystoscopy in the detection of BCa. While VUC has a specificity of >93%, its sensitivity is quite dismal at 25–40%, especially for low-grade and low-stage tumors [1], [2]. The need for more accurate urinary biomarkers for the detection of BCa is evident. A number of molecular tests have been developed to detect bladder tumors, including bladder tumor antigen (BTA) [3], nuclear matrix protein 22 (NMP-22) [4], ImmunoCyt and Urovysion [5]. Unfortunately due to their limited sensitivity and/or specificity, none of these assays have been proven accurate enough to replace cystoscopy or VUC. Thus there are no urine-based BCa tests that dominate the field to date. The inadequate power of single biomarkers may partly explain why detecting BCa using urinalysis remains a challenge. There needs to be an evolution towards tests that monitor multiple biomarkers in order to achieve the desired diagnostic accuracy.

The advent of new high-throughput genomic and proteomic techniques is driving biomarker discovery forward, and we have employed these techniques in a series of experiments [6], [7], [8] from which we have derived both nucleic acid and protein biomarker panels that facilitate non-invasive detection of BCa with unprecedented accuracy. Further selection and validation of 14 proteins from the proteomic studies, and some protein products from the genomic signature, was based on statistical ranking of association with BCa, and the availability of commercial ELISA kits. In this study, we investigated whether the monitoring of some of these targets using immune-based detection assays could confirm the potential clinical utility of a non-invasive diagnostic test. Using ELISA assays, we monitored three target proteins (C-C motif chemokine 18, CCL18; Plasminogen Activator Inhibitor 1, PAI-1; and CD44) in urine samples from a cohort of 127 subjects, and compared diagnostic performance to that obtained using the commercial BTA-Trak assay and VUC.

## Results

**Figure 1:**
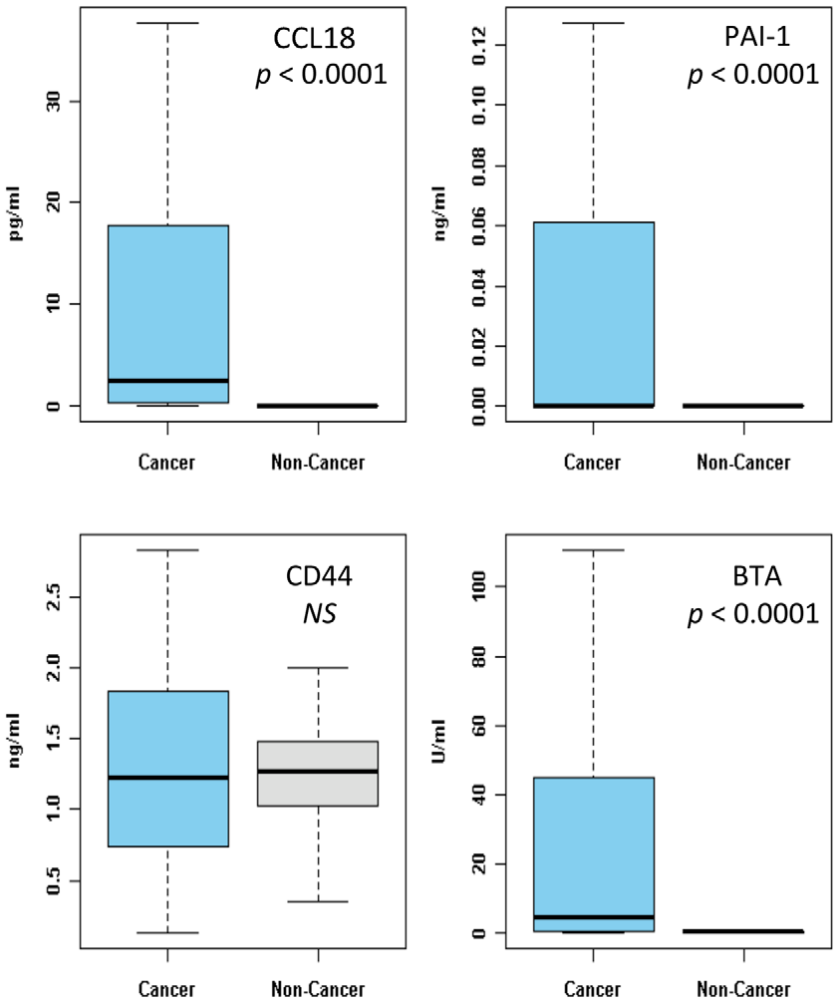
Comparison of urine concentrations of CCL18, PAI-1, CD44 and BTA between the bladder cancer and non-cancer groups. Data are normalized to urinary creatinine. Median levels are depicted by horizontal lines. Significance (*p*<0.05) was assessed by the Wilcoxon rank sum test.

Demographic, clinical and pathologic characteristics of the cohort are presented in **Table 1**. Only 28% of the cancer cohort had a positive VUC whereas VUC specificity was 98%. Urinary CCL18 and PAI-1 were undetectable in the majority of subjects with no evidence of BCa. Both BTA and CD44 were detected in samples from both tumor-bearing and control groups. Mean urinary levels (**Table 2**) of CCL18 (637.39 pg/ml vs. 4.81 pg/ml, *p* < 0.0001), PAI-1 (6.82 ng/ml vs. 0.06 ng/ml, *p*<0.0001), and BTA 1630.55 U/ml vs. 14.54 U/ml, *p*<0.0001) were significantly higher in subjects with BCa compared to subjects with no evidence of BCa. Mean urinary CD44 was elevated in subjects without BCa compared to subjects with BCa (117.22 ng/ml vs. 53.09 ng/ml, *p*<0.0001). ELISA data are presented in a boxplot figure (**Figure** **1**).

**Table 1:** Demographic and clinicopathologic characteristics of the study cohort.

	Non-cancer (%)N = 63	Cancer (%)N = 64
Median Age (range, y)	60 (30–81)	69.5 (22–90)
Male: Female ratio	55: 8	55: 9
**Race**		
White	41 (65)	58 (91)
African American	8 (13)	0 (0)
Other	14 (22)	6 (9)
Tobacco use	25 (40)	54 (84)
Gross hematuria	0 (0)	47 (73)
Suspicious/positive cytology	1 (2)	18 (28)
Median follow-up (months)	11.5	12.0
**Clinical stage**		
Tiŝ	n/a	6 (9)
Ta	n/a	15 (23)
T1	n/a	9 (14)
T2	n/a	31 (48)
T3	n/a	4 (6)
T4	n/a	2 (3)
N+ ∼	n/a	3 (5)
**Grade**		
Low	n/a	9 (14)
High	n/a	55 (86)

∧ , 4 subjects with concomitant Tis had T1 (n = 2) and T2 (n = 2) disease.

∼, Subjects with T2 (n = 1), T3 ( = 1) and T4 (n = 1) disease and node positive.

**Table 2:** Concentration of the biomarker proteins in voided urine.

	Non-cancer (%)N = 63	Cancer (%)N = 64
Urinary Proteins	Mean (range)	Mean (range)
CCl-18 (pg/ml)	4.81 (0–37.69)	637.39 (0–9523.04)
PAI-1 (ng/ml)	0.06 (0–0.64)	6.82 (0–125.26)
CD44 (ng/ml)	117.22 (16.08–616.3)	28.73 (16.67–344.04)
BTA (U/ml)	14.54 (0.5–36.87)	1630.55 (0–24865.4)

**Figure 2:**
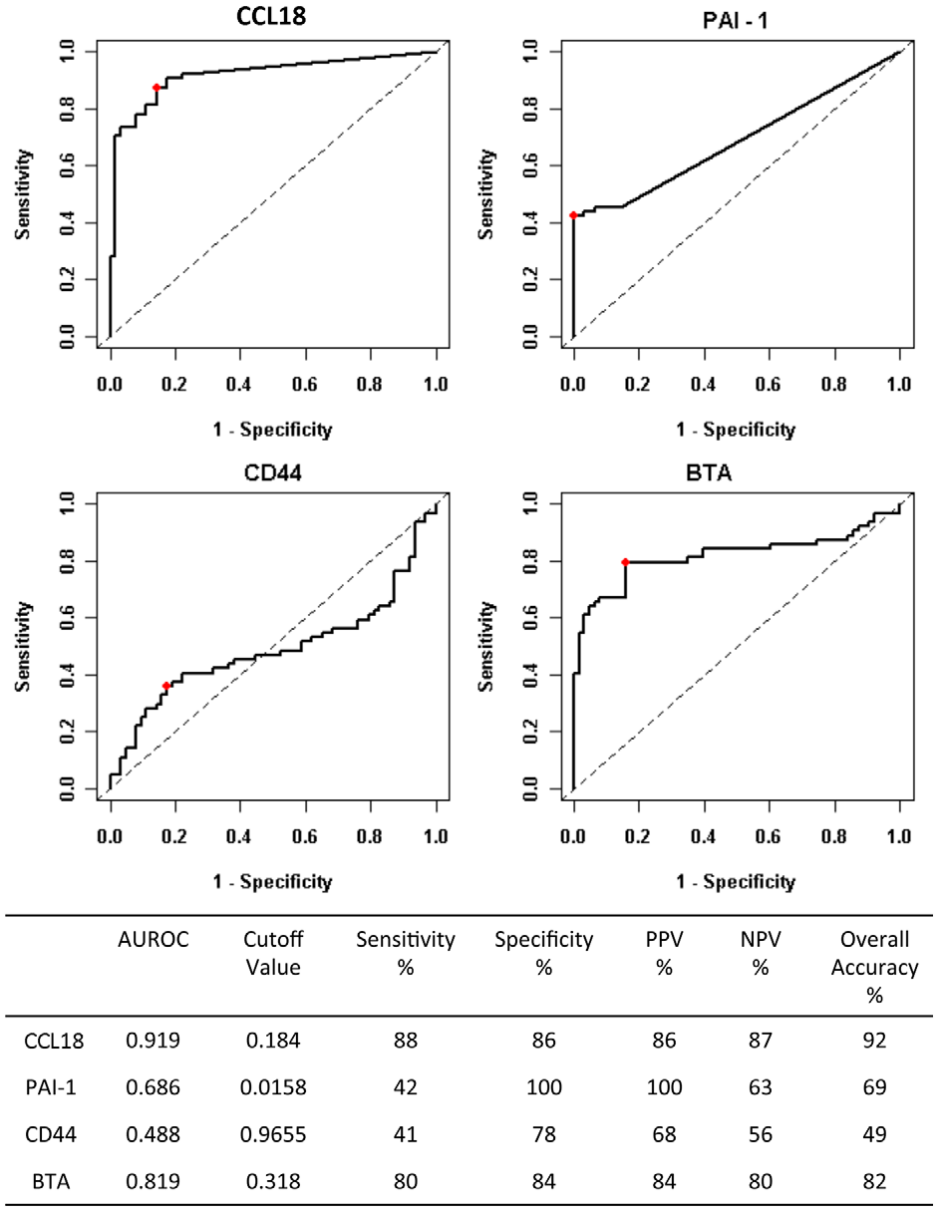
Receiver operating characteristic (ROC) curves for urinary CCL18, PAI-1, CD44 and BTA. Based on the area under the ROC curve (AUROC), Youden Index cutoff values that maximized the sum of sensitivity and specificity were determined for each biomarker (diamond). Table provides performance values for each biomarker.

The ability of the test biomarkers to predict the presence of BCa was analyzed using nonparametric ROC analyses, according to National Cancer Institute guidelines [9]. Based on the area under the ROC curve (AUROC), we determined Youden Index cutoff values to maximize the sum of sensitivity and specificity. Urinary CCL18 was the most accurate biomarker with an area under the curve of 0.919 (95% CI: 0.8704-0.9674). Using the Youden Index cutoff value (**Figure** **2**), urinary CCL18 provided a sensitivity of 88%, specificity of 86%, positive predictive value of 86% and negative predictive value of 87%. PAI-1 was a less accurate biomarker for BCa detection (area under the curve: 0.686; 95% CI: 0.6119-0.7601). Using the Youden Index cutoff value (**Figure** **2**), urinary PAI-1 analyses revealed a sensitivity of 42%, specificity of 100%, positive predictive value of 100% and negative predictive value of 63%. Elevated levels of urinary CD44 were not indicative of BCa (area under the curve: 0.488; 95% CI: 0.383-0.5937). BTA served as our positive reference assay and was noted to have an AUROC of 0.818 (95% CI: 0.74-0.90, **Figure** **2**). Using the Youden Index cutoff value, urinary BTA provided a sensitivity of 80%, specificity of 84%, positive predictive value of 84% and negative predictive value of 80%.

In multivariate logistic regression analysis that adjusted for the effects of age and race, elevated CCL18 (OR: 18.31; 95% CI: 4.95-67.70; *p*<0.0001), elevated BTA (OR: 6.43; 95% CI: 1.86-22.21; *p* = 0.0033) and reduced urinary levels of CD44 (OR: 0.039; 95% CI: 0.004-0.35; *p* = 0.0036) were associated with BCa in voided urine samples (**Table** **3**).

**Figure 3:**
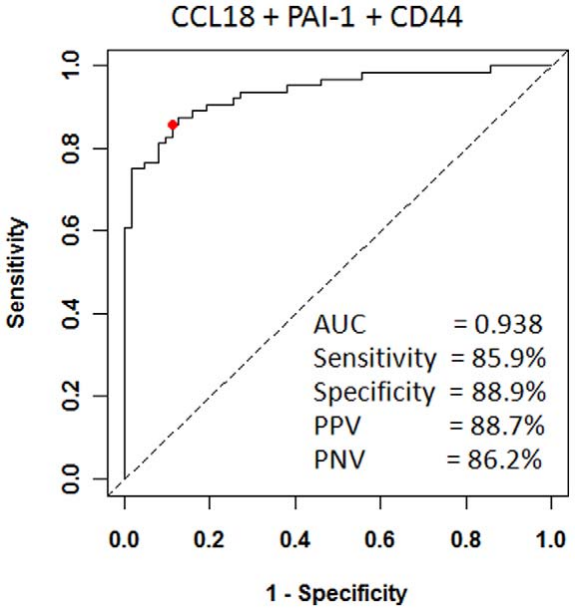
Receiver operating characteristic (ROC) curve to plot the performance of the combination of CCL18, PAI-1 and CD44 biomarkers. Based on the area under the ROC curve (AUROC), Youden Index cutoff values that maximized the sum of sensitivity and specificity were determined. Data within the figure provide performance values. PPV, positive predictive value. NPV, negative predictive value.

As stated earlier, single biomarkers fall short by not taking into consideration the complexity of heterogeneous tumors, thus a multiplex assay to detect a panel of biomarkers may prove to be beneficial. Utilizing the Youden Index cutoff values for CCL18, PAI-1 and CD44, these biomarkers were combined and analyzed (**Figure** **3**). The combination of CCL18, PAI-1 and CD44 (area under the curve: 0.938) achieved a sensitivity of 86%, specificity of 89%, positive predictive value of 89% and negative predictive value of 86%.

## Discussion

Using novel approaches [6]–[8], we have identified a preliminary diagnostic signature of 14 biomarkers related to BCa. In this study, we report our findings of three of these 14 biomarkers.

**Table 3:** Logistic regression analysis of biomarkers in voided urine.

Variable	Coefficient	Odds Ratio	95% C.I.	*p*-value
CCL18	2.91	18.31	4.95–67.70	<0.0001
CD44	−3.25	0.039	0.004–0.35	0.0036
PAI-1	−0.72	0.48	0.096–2.44	0.38
BTA	1.86	6.43	1.86–22.21	0.0033

Increased urinary levels of CCL18, PAI-1, and BTA, were significantly associated with the presence of BCa. In univariate analyses, CCL18 proved to be the marker most associated with BCa (AUC; 0.919; 95% CI, 0.8704-0.9674), with an overall positive predictive value of 86%, and a negative predictive value of 87%. Multivariate regression analysis highlighted CCL18 (OR; 18.31; 95% CI, 4.95-67.70, *p*<0.0001), BTA-Trak© (OR; 6.43; 95% CI, 1.86-22.21, *p* = 0.0033) and CD44 (OR; 0.039; 95% CI, 0.004-0.35, p = 0.0036) as independent variables in the detection of BCa in voided urine samples. With an overall accuracy of 92%, CCL18 clearly outperformed the other biomarkers, including BTA, as a target protein for BCa detection by urinalysis. The inclusion of additional markers (e.g., PAI-1 and CD44) to a multiplex panel may not increase the predictive power significantly (AUC 0.919 vs. 0.938), but their addition may make the model more robust to errors thus illustrating the benefit of a multiplex assay to diagnose BCa.

CCL18 is a member of the cytokine family of secreted proteins involved in immunoregulatory and inflammatory processes. CCL18 is thought to promote the invasiveness of cancer cells by triggering integrin clustering and enhancing their adherence to the extracellular matrix, and a receptor (PITPNM3) for this cytokine has been recently identified [10]. A range of cytokines and their receptors are known to be aberrantly expressed in tumor cells and so it is conceivable that CCL18 can act in a paracrine fashion during tumor initiation/establishment. As a biomarker of malignant disease, CCL18 has been reported to be indicative of ovarian [11], [12] and cervical cancer [13]. A proteomics approach identified elevated serum CCL18 as correlated with ovarian cancer, and this finding was validated using ELISA in 535 samples. In combination with CXCL1, CCL18 monitoring outperformed CA125 as a circulating ovarian cancer biomarker [12]. A study analyzing differential gene expression in 33 solid tissue samples identified CCL18 as one of twenty transcripts significantly over-expressed in invasive cervical cancers [13].

PAI-1 is a member of the serine proteinase inhibitor super family and is the principal inhibitor of tissue plasminogen activator (tPA) and urokinase (uPA), thus it regulates fibrinolysis [14]. PAI-1 is considered to be a multifunctional protein, which can also modulate tumor cell growth and migration, angiogenesis and cell adhesion [15], [16]. Results from different human cancers have demonstrated that the levels of tPA or uPA are significantly higher in cancerous tissue relative to corresponding normal tissue [17]–[19]. Hudson *et* *al.* reported that both non-invasive and invasive human BCa cell lines produce PAI-1 [20], and elevated PAI-1 gene and protein expression in tissues and plasma has been observed in BCA patients with a poor prognosis [21]. In a study monitoring PAI-1 in tissues, serum and urine, significantly higher levels in tissues and serum correlated with poor prognosis, but this was not true for urinary PAI-1 [21]. In our study, we were looking for the presence of BCa, and did not consider prognosis. We were able to demonstrate that urinary PAI-1 was significantly elevated in subjects with BCa (6.82 vs. 0.06, *p*<0.0001), however, in multivariate analysis, PAI-1 was not an independent predictor of BCa. PAI-1 may not be a powerful stand-alone biomarker for BCa detection, but further investigation is needed to determine if it adds value to multiple biomarker panels.

Many studies have investigated CD44 as a biomarker for cancer, including BCa. CD44 is a ubiquitously expressed transmembrane glycoprotein that can interact with a variety of ligands, e.g., hyaluronic acid synthases, and is involved in cell-cell interactions, cell adhesion and migration [22]. Transcripts for this gene undergo complex alternative splicing resulting in a myriad of mRNA and protein isoforms with functional diversity, and this makes it difficult to achieve consensus on which CD44 targets are valid biomarkers. Klatte *et* *al.* demonstrated that absent CD44v6 expression in excised tissue is an independent adverse predictor of BCa recurrence and overall survival [23], and over-expression of CD44V8-10 in exfoliated urothelial cells has been reported to be an independent prognostic predictor in patients with BCa [24]. We previously developed a sandwich-ELISA assay that measured the CD44v6 protein isoform protein in urothelial cell lysates. In a study of 65 cases, the sandwich-ELISA assay achieved 81% sensitivity and 100% specificity for BCa diagnosis [25]. In line with those findings, a study was able to associate soluble CD44v6 mRNA in cancer patient urine with BCa [26]. Our urothelial cell profiling and validation study [7] confirm that monitoring CD44 transcripts may have value in clinical evaluation, but soluble urinary CD44 protein does not seem to be a promising diagnostic biomarker.

We recognize that our study has several limitations. First as a tertiary care facility, we tend to see more high-grade, high-stage disease, which is reflected in our study cohort. To confirm the robustness of CCL18, subsequent studies must assess larger cohorts that include subjects with low-grade, low-stage disease. Second, processed, banked urines were analyzed. Urines were centrifuged and separated into cellular pellet and supernatant prior to storage at −80°C. It is feasible that freshly voided urine samples may provide different results, and it is fresh urine that would be the material used for point-of-care assays. We are currently investigating the performance of selected biomarkers in urines processed via a number of different protocols, including freshly voided urines. Next, the sensitivity of VUC in our cohort of predominantly high-grade (grade 3) disease (28%) was lower than would be expected. This calls into question the known inter-observer variability of interpreting VUC. In subsequent studies, we will utilize two cytopathologists to interpret these results. Furthermore, it is uncertain how the protein composition of the urine supernatant may change during frozen storage. The number of freeze-thaw cycles was kept to 1–2 by dividing the urine supernatant into multiple small aliquots. Lastly, our sample size of 127 is small and the two groups that comprised the 127 subjects were relatively homogeneous, i.e. either active cancer, or control cases with no active cancer, no history of cancer, no urinary tract infection, no urolithiasis, and no gross hematuria. Thus we were not able to assess sensitivity/specificity of our biomarkers among different stages/grades. The specificity of promising biomarkers such as CCL18 needs to be tested in cohorts that are known to be problematic with other urine-based assays (e.g., hematuria, urinary tract infection, stones and voiding dysfunction). As we refine our diagnostic molecular signatures and select specific targets for nucleic acid-based or immune-based assay development, our findings will be confirmed in large, prospective and multicenter collaborative phase 2 and 3 trials consisting of subjects not only with BCa, but with urinary tract infection, urolithiasis, gross hematuria and voiding symptoms.

The development of urine-based bladder cancer biomarkers would be of tremendous benefit to both patients and healthcare systems. We have demonstrated in a preliminary study that elevated urinary concentration of CCL18 protein is strongly associated with the presence of BCa. Furthermore, the combination of CCL18, PAI-1 and CD44 may not increase the predictive power of CCL18 significantly, but may make the model more robust to errors. Larger, prospective studies are needed to determine the potential role of CCL18 in the evaluation of patients who are at risk of BCa.

## Materials and Methods

### Patients and specimen processing

Under MD Anderson Cancer Center Orlando Institutional Review Board approval and informed written consent, voided urine samples, and associated clinical information were prospectively collected. The study cohort consisted of 63 individuals with no previous history of urothelia carcinoma, gross hematuria, active urinary tract infection or urolithiasis, and 64 individuals with newly diagnosed urothelial carcinoma. Patients with known renal disease or documented renal insufficiency were not enrolled. According to the International Consensus Panel on Bladder Tumor Markers [27], this cohort served as a phase II (validation study). Data is reported using the STARD criteria [28]. In our cancer group, axial imaging of the abdomen and pelvis and cystoscopy were performed, and urothelial cell carcinoma was confirmed by histological examination of excised tissue. Pertinent information on clinical presentation, staging, histologic grading [29], [30] and outcome were recorded (**Table** **1**).

Prior to any type of therapeutic intervention, 50–100 mL of voided urine was obtained from each subject. Fifty milliliters of urine was used for clinical laboratory analyses per standard procedures. The remaining urine aliquot was assigned a unique identifying number before immediate laboratory processing. Each urine sample was centrifuged at 600× g 4°C for 5 min. The supernatant was decanted and aliquoted, while the urinary pellet was snap frozen. Both the supernatant and pellet were stored at −80°C prior to analysis. Aliquots of urine supernatants were thawed and analyzed for protein content using a Pierce 660-nm Protein Assay Kit (Thermo Fisher Scientific Inc., Waltham, MA, USA). Protein concentrations were measured using the NanoDrop spectrophotometer (ND-1000, ThermoScientific, Wilmington, DE, USA).

### Urine Based Enzyme-Linked Immunosorbent Assay (ELISA)

Levels of human C-C motif chemokine 18 (CCL18), also known as CCL18 (Cat # ab100620 Abcam, Cambridge, MA, USA), human Plasminogen Activator Inhibitor 1 (PAI-1, Cat# EA-0207 Signosis Inc., Sunnyvale, CA, USA), and CD44 (Cat # ab 45912 Abcam, Cambridge, MA, USA) were monitored in urine samples using enzyme-linked immunosorbent assay. Commercially available ELISA assays were also used to measure levels of urinary hemoglobin (Cat#E88-135 Bethyl Laboratories Inc., Montgomery, TX, USA), and BTA (BTA-Trak© Ca# 662150 Polymedco Inc. Cortlandt Manor, NY, USA). Readers of these assays were blinded as to disease status. All assays were conducted according to manufacturer's instructions. Calibration curves were prepared using purified standards for each protein assessed. Curve fitting was accomplished by either linear or four-parameter logistic regression following manufacturer's instructions.

### Creatinine Assay

Creatine is converted non-enzymatically to the metabolite creatinine, which diffuses into the blood and is excreted into the urine by the kidneys at a constant rate. Consequently, urinary creatinine is a useful tool for normalizing the levels of other molecules found in urine [31], [32]. Our preliminary results (not shown) demonstrated that due to the hematuria (microscopic or gross blood in the urine), which contains elevated levels of proteins, protein would not be a good marker for normalization. Thus, the concentrations of all monitored proteins (CCL18, PAI-1, CD44 and BTA) were normalized to urinary creatinine and these concentrations were reported as a ratio relative to urinary creatinine values. The creatinine assay was conducted according to the manufacturer's instructions (Cat# KGE005 R&D Systems Inc., Minneapolis, MN, USA). Briefly, urine supernatants were thawed, diluted with distilled water and treated with alkaline picrate solution. Treated samples were measured on a microplate reader (Bio-tek, Synergy^TM^ HT, VT) at a wavelength of 490 nm. A standard curve using purified standards was generated by regression analysis using four-parameter logistic curve-fit, and signal intensities were converted to concentrations.

### Statistical Analysis

The association between each biomarker and BCa was tested using the Wilcoxon rank sum test. Nonparametric receiver operating characteristic (ROC) curves in which the value for sensitivity is plotted against false-positive rate (1-specificity) were generated. We defined a diagnostic test as positive or negative for BCa detection using a cutoff value. The optimal cutoff (Youden index) was selected to maximize the sum of the sensitivity and specificity [33]. The accuracy of a biomarker to predict the presence of BCa was defined as the average of the sensitivity and the specificity. To assess the independent association between biomarkers and BCa, logistic regression analysis was performed with BCa status (yes vs. no) as the response variable, and CCL18, PAI-1, CD44, BTA concentrations as the explanatory variables. Statistical significance in this study was set at *p*<0.05 and all reported *p* values were 2-sided. All analyses were performed with SAS software version 9.1.3.

## References

[1] Rife CC, Farrow GM, Utz DC (1979). Urine cytology of transitional cell neoplasms.. Urol Clin North Am.

[2] Cajulis RS, Haines GK, 3rd, Frias-Hidvegi D, McVary K, Bacus JW (1995). Cytology, flow cytometry, image analysis, and interphase cytogenetics by fluorescence in situ hybridization in the diagnosis of transitional cell carcinoma in bladder washes: a comparative study.. Diagn Cytopathol 13: 214–223; discussion.

[3] Gutiérrez Baños JL, del Henar Rebollo Rodrigo M, Antolín Juárez FM, García BM (2001). Usefulness of the BTA STAT Test for the diagnosis of bladder cancer.. Urology.

[4] Hwang EC, Choi HS, Jung SI, Kwon DD, Park K (2010). Use of the NMP22 BladderChek Test in the Diagnosis and Follow-Up of Urothelial Cancer: A Cross-sectional Study..

[5] Daniely M, Rona R, Kaplan T, Olsfanger S, Elboim L (2007). Combined morphologic and fluorescence in situ hybridization analysis of voided urine samples for the detection and follow-up of bladder cancer in patients with benign urine cytology.. Cancer.

[6] Kreunin P, Zhao J, Rosser C, Urquidi V, Lubman DM, Goodison S (2007). Bladder cancer associated glycoprotein signatures revealed by urinary proteomic profiling.. *J Proteome Res*;.

[7] Yang N, Feng S, Shedden K, Xie X, Liu Y (2011). Urinary Glycoprotein Biomarker Discovery for Bladder Cancer Detection using LC-MS/MS and Label-free Quantification.. Clin Cancer Res.

[8] Rosser CJ, Liu L, Sun Y, Villicana P, McCullers M (2009). Bladder cancer-associated gene expression signatures identified by profiling of exfoliated urothelia.. Cancer Epidemiol Biomarkers Prev.

[9] Pepe MS, Feng Z, Janes H, Bossuyt PM, Potter JD (2008). Pivotal evaluation of the accuracy of a biomarker used for classification or prediction: standards for study design.. J Natl Cancer Inst.

[10] Chen J, Yao Y, Gong C, Yu F, Su S (2011). CCL18 from Tumor-Associated Macrophages Promotes Breast Cancer Metastasis via PITPNM3.. Cancer Cell.

[11] Zohny SF, Fayed ST (2010). Clinical utility of circulating matrix metalloproteinase-7 (MMP-7), CC chemokine ligand 18 (CCL18) and CC chemokine ligand 11 (CCL11) as markers for diagnosis of epithelial ovarian cancer.. Med Oncol.

[12] Wang Q, Li D, Zhang W, Tang B, Li QQ (2011). Evaluation of proteomics-identified CCL18 and CXCL1 as circulating tumor markers for differential diagnosis between ovarian carcinomas and benign pelvic masses..

[13] Rajkumar T, Sabitha K, Vijayalakshmi N, Shirley S, Bose MV (2011). Identification and validation of genes involved in cervical tumourigenesis.. BMC Cancer.

[14] Schmitt M, Mengele K, Napieralski R, Magdolen V, Reuning U (2010). Clinical utility of level-of-evidence-1 disease forecast cancer biomarkers uPA and its inhibitor PAI-1.. Expert Rev Mol Diagn.

[15] Duffy MJ (1996). Proteases as prognostic markers in cancer.. Clin Cancer Res.

[16] McMahon GA, Petitclerc E, Stefansson S, Smith E, Wong MK (2001). Plasminogen activator inhibitor-1 regulates tumor growth and angiogenesis.. J Biol Chem.

[17] Span PN, Witjes JA, Grebenchtchikov N, Geurts-Moespot A, Moonen PM (2008). Components of the plasminogen activator system and their complexes in renal cell and bladder cancer: comparison between normal and matched cancerous tissues.. BJU Int.

[18] Malinowsky K, Bollner C, Hipp S, Berg D, Schmitt M (2010). UPA and PAI-1 analysis from fixed tissues – new perspectives for a known set of predictive markers.. Curr Med Chem.

[19] Pedersen H, Grondahl-Hansen J, Francis D, Osterlind K, Hansen HH (1994). Urokinase and plasminogen activator inhibitor type 1 in pulmonary adenocarcinoma.. Cancer Res.

[20] Hudson MA, McReynolds LM (1997). Urokinase and the urokinase receptor: association with in vitro invasiveness of human bladder cancer cell lines.. J Natl Cancer Inst.

[21] Becker M, Szarvas T, Wittschier M, vom Dorp F, Totsch M (2010). Prognostic impact of plasminogen activator inhibitor type 1 expression in bladder cancer.. Cancer.

[22] Golshani R, Lopez L, Estrella V, Kramer M, Iida N (2008). Hyaluronic acid synthase-1 expression regulates bladder cancer growth, invasion, and angiogenesis through CD44.. Cancer Res.

[23] Klatte T, Seligson DB, Rao JY, Yu H, de Martino M (2010). Absent CD44v6 expression is an independent predictor of poor urothelial bladder cancer outcome.. J Urol.

[24] Miyake H, Eto H, Arakawa S, Kamidono S, Hara I (2002). Over expression of CD44V8-10 in urinary exfoliated cells as an independent prognostic predictor in patients with urothelial cancer.. J Urol.

[25] Woodman AC, Goodison S, Drake M, Noble J, Tarin D (2000). Noninvasive diagnosis of bladder carcinoma by enzyme-linked immunosorbent assay detection of CD44 isoforms in exfoliated urothelia.. Clin Cancer Res.

[26] Eissa S, Zohny SF, Swellam M, Mahmoud MH, El-Zayat TM (2008). Comparison of CD44 and cytokeratin 20 mRNA in voided urine samples as diagnostic tools for bladder cancer.. Clin Biochem.

[27] Lokeshwar VB, Habuchi T, Grossman HB, Murphy WM, Hautmann SH (2005). Bladder tumor markers beyond cytology: International Consensus Panel on bladder tumor markers.. Urology.

[28] Bossuyt PM, Reitsma JB, Bruns DE, Gatsonis CA, Glasziou PP, Irwig LM, Lijmer JG, Noher D, Rennie D, de Vet HCW, for the STARD group. Towards complete and accurate reporting of studies of diagnostic accuracy: the STARD initative.. *Family Practice* 2004,.

[29] Greene FL, ACS AJCCCancerStagingManual;AmericanJointCommitteeonCancer (2002). American Joint Committee on Cancer, American Cancer Society.. editor.

[30] Montironi R, Lopez-Beltran A (2005). The 2004 WHO classification of bladder tumors: a summary and commentary.. Int J Surg Pathol.

[31] Pisitkun T, Johnstone R, Knepper MA (2006). Discovery of urinary biomarkers.. Molecular & Cellular Proteomics.

[32] Pu FS (1992). Urinary excretion of creatinine in normal subjects.. Chinese Pharmaceutical Journal.

[33] Fluss R, Faraggi D, Reiser B (2005). Estimation of the Youden Index and its associated cutoff point.. Biom J.

